# Early elevated neutrophil‐to‐lymphocyte ratio associated with remote diffusion‐weighted imaging lesions in acute intracerebral hemorrhage

**DOI:** 10.1111/cns.13249

**Published:** 2019-10-25

**Authors:** Jia‐Wen Li, Yu‐Yu Xu, Ye‐Jun Chen, Wei‐Wei Fan, Xu‐Hua Xu, Jin‐Song Cai, Lu‐Sha Tong, Feng Gao

**Affiliations:** ^1^ Department of Neurology The Second Affiliated Hospital School of Medicine Zhejiang University Hangzhou China; ^2^ Department of Neurology The Fourth Affiliated Hospital School of Medicine Zhejiang University Yiwu China; ^3^ Department of Radiology The Second Affiliated Hospital School of Medicine Zhejiang University Hangzhou China

**Keywords:** biomarker, diffusion‐weighted imaging (DWI), intracerebral haemorrhage (ICH), neutrophil‐to‐lymphocyte ratio (NLR)

## Abstract

**Aims:**

To explore the relationship between the circulating neutrophil‐to‐lymphocyte ratio (NLR) and the remote diffusion‐weighted imaging lesions (R‐DWILs) after spontaneous intracerebral hemorrhage (ICH).

**Methods:**

Consecutive patients with spontaneous ICH were prospectively collected from November 2016 to May 2018 and retrospectively analyzed. We included subjects who presented within 24 hours after symptom onset and were free of detectable infections on admission or in hospital. Blood samples were obtained at 24‐48 hours after ICH ictus, while all complete MRI scans were performed at 5‐8 days. R‐DWILs were defined as focal hyperintensities remote from the site of the ICH or the peri‐hematoma regions. NLR was calculated by dividing the absolute neutrophil counts by the absolute lymphocyte counts. Multivariate binary logistic regression models were generated to evaluate the relationship between NLR and R‐DWILs.

**Results:**

One hundred sixty‐three subjects met eligibility criteria (age 62.3 ± 13.6 years, 60.7% males), of whom 31(19.0%) experienced R‐DWILs. Higher circulating NLR was documented in patients with R‐DWILs. With the best cutoff value of 6.01, elevated NLR was independently associated with the presence of R‐DWILs (OR = 3.170, 95% CI 1.306‐7.697, *P* = .011) in the bivariate logistic regression analysis with adjustment for age, sex, atrial fibrillation, previous ischemic stroke/TIA, SBP on admission, hematoma volume, and IVH.

**Conclusions:**

This study provides significant evidence of the association between circulating NLR and R‐DWILs in spontaneous ICH patients. Patients with NLR > 6.01 at 24‐48 hours after ICH ictus should be paid more attention to when evaluating R‐DWILs.

## 1 INTRODUCTION

Intracerebral hemorrhage (ICH) accounts for 6.5%‐19.6% of cases of stroke. Despite advances in neurocritical care, it remains a devastating disease for high morbidity, mortality, and recurrence rate among survivors.^1^ The predictive factors for the detrimental outcome are increasing age, clinical severity neurological grade at initial presentation, hematoma volume, infratentorial location of ICH, and intraventricular extension.^2^, ^3^, ^4^ Moreover, the presence of intracerebral remote diffusion‐weighted imaging lesions (R‐DWILs) has been known to worsen the stroke outcome.^5^ R‐DWILs are defined as focal hyperintensities remote from the site of the ICH or the peri‐hematoma region on diffusion‐weighted images, which are detected in about 11%‐41% of ICH patients.^6^, ^7^, ^8^, ^9^ It is an essential complication that requires attention owing to its significant effect on the increased risk of recurrent ICH, cognitive impairment, and mortality.

The exact pathophysiology of R‐DWILs in ICH patients remains uncertain. Apart from the progression of microangiopathy, inflammatory response following ICH has also been implicated to exert influence on the development of R‐DWILs.^5^, ^10^ However, prior studies examining the effect of inflammation, in the form of leukocytes, had failed to find a correlation with R‐DWILs.^8^, ^11^ It was possible that they analyzed leukocytes as an entirety instead of a subset and neglected the respective implications of neutrophils or lymphocytes following ICH. Recently, the neutrophil‐to‐lymphocyte ratio (NLR) emerges as an indicator of inflammatory status in patients with ST‐segment elevation myocardial infarction, cerebral venous sinus thrombosis, and ischemic stroke.^12^, ^13^, ^14^ It integrates neutrophils and lymphocytes and reflects the shift between them. In terms of ICH, NLR has been shown to be better than leukocytes alone for predicting peri‐hematoma edema growth^15^ and adverse clinical outcomes.^16^, ^17^

The primary aim of this study was to analyze the relationship between the circulating NLR and the R‐DWILs in patients of ICH. Our findings showed that patients with an elevated NLR in acute stage should be intensely noted for the increased risk of R‐DWILs.

## 2 METHODS

### 2.1 Participants

**Figure 1:**
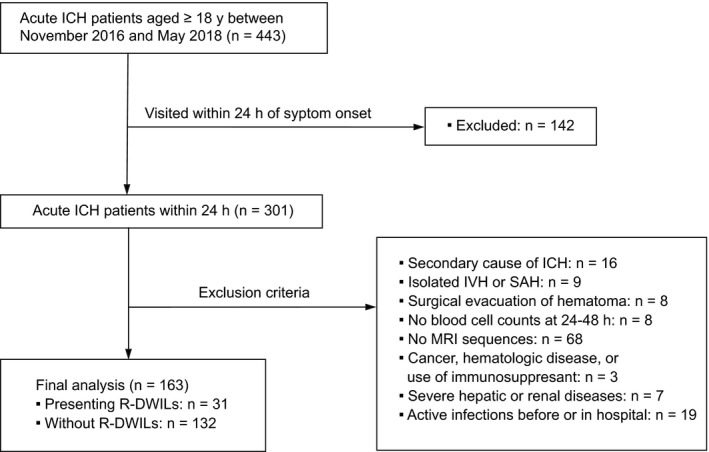
Section flowchart of this cohort

We prospectively collected a consecutive series of patients with ICH admitted to the Department of Neurology of Second Affiliated Hospital of Zhejiang University between November 2016 and May 2018. Criteria for inclusion were as follows: age ≥ 18 years; admitted within 24 hours of initial symptom onset (onset was defined at the time a patient was last known to be without stroke symptoms); verified diagnosis of ICH by CT scan. Criteria for exclusion were as follows: (a) secondary causes of ICH(n = 16) such as hemorrhagic transformation of ischemic stroke, aneurysmal, cavernomas, arteriovenous malformations, central venous thrombosis, trauma‐related, or tumor; (b) isolated intraventricular hemorrhage or subarachnoid hemorrhage(n = 9); (c) surgical evacuation of hematoma(n = 8); (d) unavailability to get complete blood cell samples(n = 8); and (e) presenting contraindications or refusal to MRI(n = 68). As NLR was significantly affected by severe systemic inflammation,^18^ participants with a history of malignant tumor, hematologic disease, receiving permanent immunomodulatory treatment (eg, corticosteroids, methotrexate, other cytostatic drugs, and biologicals) (n = 3), or presenting severe hepatic or renal diseases (n = 7) were excluded from our cohort. Cases were also extracted who suffered from active infections within 2 weeks before admission or diagnosed in the hospital (n = 19). The precise definition of infection was based on the Centers for Disease Control and Prevention (CDC)/ National Healthcare Safety Network (NHSN) criteria. The patient flowchart of this cohort was summarized in Figure 1. Ultimately, a total of 163 patients remained for analysis.

### 2.2 Data collection

Demographic information and clinical history were retrieved through patients or family members’ interviews. We collected age, sex, smoking and alcohol drinking history, and comorbid conditions such as the history of hypertension, diabetes mellitus, atrial fibrillation, previous ICH, or previous ischemic stroke/TIA. Prestroke medications such as antiplatelet agents, antihypertensive drugs, and hypoglycemic treatments were also collected. Hypertension was defined by the known elevation of blood pressure on at least 2 separate occasions according to the medical history or the use of antihypertensive medications. Diabetes mellitus was described by the patients had been informed of this diagnosis by a physician before admission or was receiving hypoglycemic treatments (dietary, oral antidiabetic agents, or insulin) or patients who with serum hemoglobin A1c levels ≥ 6.5%. Previous ICH ischemic stroke/TIA was defined as a history of related syndromes or documented information in the medical record. Smoking and alcohol drinking status was ascertained according to medical history. Moreover, initial evaluation parameters (systolic blood pressure [SBP], diastolic blood pressure [DBP], Glasgow Coma Scale [GCS], and National Institute of Health Stroke Scale [NIHSS]) on admission were also collected with well‐trained neurologists and noted for analysis.

### 2.3 Laboratory variables

Peripheral complete blood samples were obtained at 24‐48 hours after ICH onset in all participants, and the blood samples were immediately centrifuged (2264 *g* for 10 minutes at 37°C) after being collected in a calcium EDTA tube. Total leukocytes, neutrophil, and lymphocyte counts were determined using an autoanalyzer (XN‐9000, Sysmex). NLR was computed by dividing the absolute neutrophil counts by the absolute lymphocyte counts. Other hematologic and biochemical data—fasting blood glucose, erythrocyte sedimentation rate (ESR), and high sensitive C‐reactive protein (hs‐CRP)—were obtained together at 24‐48 hours.

### 2.4 Neuroradiology variables

All patients underwent a baseline CT brain to verify spontaneous ICH and the initial CT brain scans after admission were reviewed for analysis. The site of hematoma was categorized as lobar, deep, brainstem, or cerebellum, and the volume of hematoma was measured using the ABC/2 method.^19^ IVH was defined as an intraventricular hyperdense image not attributable to calcification or the choroid plexus.

**Figure 2:**
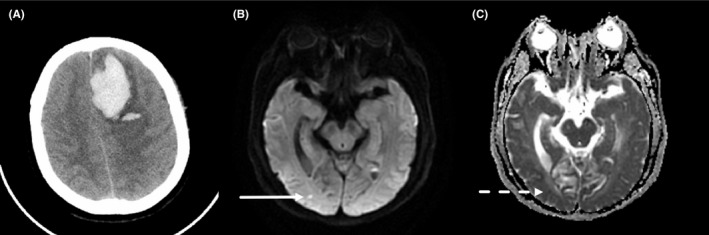
In a 43‐y‐old man with left frontal lobe hemorrhage (A), diffusion‐weighted imaging (DWI) shows a small remote ischemic lesion on the right occipital lobe (B), with corresponding low signal intensity in apparent diffusion coefficient (ADC) map (C)

MRI was performed on 1.5‐Telsa (Sonata, Siemens) or 3.0‐Telsa scanner (Signa HDxt, GE Healthcare) with standardized protocol consisted of axial T1‐weighted, T2‐weighted, T2 FLAIR, DWI, and apparent diffusion coefficient (ADC) sequences. Axial DWI sequences were acquired on 1.5T [repetition time (TR) 3,100 ms, echo time (TE) 84 ms, b = 0/1000 s/mm^2^, 6‐mm slice thickness, 0.5‐mm gap, FOV 230 mm], or 3.0T scanner (TR 5,200 ms, TE 75 ms, b = 0/1000 s/mm^2^, 6‐mm slice thickness, 0.5‐mm gap, and FOV 240 mm) with different parameters. R‐DWILs were defined as hyperintense distinct from the focal hematoma (>20 mm) on DWI sequence (*b* = 0/1000 s/mm^2^), measuring <20 mm in diameter.^20^ The corresponding regions on the ADC map were also viewed to confirm that the diffusion coefficient was reduced relative to the adjacent nonlesional brain parenchyma (Figure 2). All images were reviewed by an experienced clinical neurologist (Lu‐sha Tong) and an experienced radiologist (Jin‐song Cai) without knowledge of clinical information. The two researchers reached high interrater reliability (Kappa = 1.0).

### 2.5 Ethical approval

Informed consent was obtained from all participants or legal representatives. And the study protocol was approved by the Ethics Review Board of Second Affiliated Hospital of Zhejiang University.

### 2.6 Statistical analysis

Patients were divided into two groups based on the presence vs absence of R‐DWILs. Kolmogorov‐Smirnov test was used to figure out the distribution pattern. Continuous variables with normal distributions were shown as the mean ± SD while other variables were presented as the median with interquartile range (IQR). Categorical data were expressed as number (proportion). Student *t‐*test or the Mann‐Whitney *U*‐test was used for continuous variables, and chi‐square analysis or the Fisher exact test for categorical variables. An exploratory evaluation for best cut‐point thresholds of NLR was performed using the receiver operating characteristic (ROC) curve analysis. To assess the association between NLR and R‐DWILs, multivariate binary logistic regression analysis was developed when controlling for confounding factors. Two kinds of covariates were entered into the multivariable models using conditional forward stepwise logistic regression: (a) those variables generated using candidate variables with univariate associations of *P* < .1 and (b) significant covariates referenced from previously published article. All statistical analyses were undertaken using SPSS version 23.0 (IBM). Two‐tailed significance values were applied, and statistical significance was defined as *P* < .05.

## 3 RESULTS

### 3.1 Clinical characteristics of the patients

Of the 442 participants with intracerebral hemorrhage, 279 met at least one exclusion criteria and 10 (2.2%) suffered death during hospitalization period. A total of 163 patients (m/w ratio, 1.55 and mean age, 62.3 ± 13.6 years) were admitted to our analysis. Majority of the patients had a history of hypertension (74.8%), and 34.4% of patients took antihypertensive drugs. Median time from ICH onset to our emergency was 6 hours (IQR, 3‐20 hours), and all patients presented relatively elevated systolic blood pressure on admission (median, 158 mm Hg, IQR, 140‐179 mm Hg). A total of 112 patients (68.7%) experienced deep‐location lesions, and the median hematoma volume was 11.4 cm^3^ (IQR, 5.6‐22.3 cm^3^). Median time to MRI scans was 6 day (IQR, 5‐8 day). The body temperature before MRI (median, 38°C, IQR, 37.5‐38.4°C) elevated in most subjects.

R‐DWILs were present in 31 (19%) patients. Table 1 showed baseline characteristics with comparison between patients with and without R‐DWILs. Most of these characteristics were comparable, but patients with R‐DWILs were a bit older (mean age, 61.0 ± 13.7 vs 68.0 ± 11.9, *P* = .005), and were found to be prone to experience IVH (61.3% vs 28.8%, *P* = .001) and elevated body temperature (38.2°C vs 37.8°C, *P* = .006). Moreover, higher NLR (7.7 vs 5.0, *P* = .001) and higher blood glucose (6.7 vs 5.8, *P* = .002) were documented in patients with R‐DWILs.

**Table 1:** Baseline clinical characteristics of the study participants, according to presence vs absence R‐DWILs

Characteristics	All included n = 163	R‐DWILs (+) n = 31	R‐DWILs (−) n = 132	*P*‐value
Demographics				
Age, y, mean ± SD	62.3 ± 13.6	68.0 ± 11.9	61.0 ± 13.7	.005**
Male, (n %)	99 (60.7)	21 (67.7)	78 (59.1)	.375
Clinical history, (n %)				
Hypertension	122 (74.8)	24 (77.4)	98 (74.2)	.741
Diabetes mellitus	18 (11)	3 (9.7)	15 (11.4)	1.000
Atrial fibrillation	8 (4.9)	1 (3.2)	7 (5.3)	1.000
Previous ischemic stroke/TIA	15 (9.2)	6 (19.4)	9 (6.8)	.068
Previous ICH	12 (7.4)	3 (9.7)	9 (6.8)	.868
Prestroke medications, (n %)				
Antiplatelet agents	9 (5.5)	4 (12.9)	5 (3.8)	.118
Antihypertensive drugs	56 (34.4)	13 (41.9)	43 (32.6)	.323
Antidiabetic drugs	8 (4.9)	1(3.2)	7(5.3)	.977
Smoker	48 (29.4)	13 (41.9)	35 (26.5)	.090
Alcohol	35 (21.5)	6 (19.4)	29 (22.0)	.750
On admission status, median [IQR]				
From onset to emergency, h	6.0 [3.0, 20.0]	5.0 [3.0, 24.0]	6.0 [3.0, 19.8]	.912
Glasgow coma scale	15.0 [13.0, 15.0]	15.0 [13.0, 15.0]	15.0 [13.0, 15.0]	.559
NIH stroke scale	7.0 [3.0, 11.0]	7.0 [3.0, 12.0]	7.5 [3.0, 11.0]	.643
SBP on admission, mm Hg	158 [140, 179]	162 [149, 179]	156 [140, 178]	.226
DBP on admission, mm Hg	89 [80, 100]	95 [82, 110]	88 [80, 99]	.102
Neuroradiological data				
Location‐lobar, (n %)	37 (22.7)	9 (29.0)	28 (21.2)	.350
Location‐deep, (n %)	112 (68.7)	20 (64.5)	92 (69.7)	.576
IVH, (n %)	57 (35)	19 (61.3)	38 (28.8)	.001**
SAH, (n %)	21 (12.9)	4 (12.9)	17 (12.9)	1.000
Hematoma volume, cm^3^, median [IQR]	11.4 [5.6, 22.3]	15.1[8.7, 29.2]	10.9[5.5, 21.0]	.157
From onset to MRI scans, d, median [IQR]	6.0 [5.0, 8.0]	6.0 [5.0, 7.0]	6.0 [5.0, 8.0]	.771
Tmax before MRI, °C, median [IQR]	38.0[37.5,38.4]	38.2[37.8,38.6]	37.8[37.5,38.4]	.006**
Laboratory values, median [IQR]				
ESR, mm/h	13.0 [7.0, 19.0]	14.0 [13.0, 23.0]	13.0 [7.0, 18.8]	.064
Leukocytes at 24‐48 h, 10^9^/L	8.7 [7.1, 11.1]	9.8 [6.8, 11.8]	8.6 [7.1, 11.0]	.509
hs‐CRP, mg/L	6.1 [3.6, 17.6]	5.3 [3.0, 11.1]	7.7 [4.5, 27.9]	.134
GLU, mmol/L	5.9 [5.1, 6.9]	6.7 [5.9, 7.5]	5.8 [5.0, 6.7]	.002**
NLR	5.5 [3.7, 8.8]	7.7 [5.5, 11.6]	5.0 [3.6, 7.8]	.001**

Abbreviations: DBP, diastolic blood pressure; ESR, erythrocyte sedimentation rate; GLU, fasting blood glucose; hs‐CRP, high sensitive c‐reactive protein; ICH, intracerebral hemorrhage; IQR, interquartile range; IVH, intraventricular hemorrhage; NLR, neutrophil‐to‐lymphocyte ratio; R‐DWILs, remote diffusion‐weighted imaging lesions; SAH, subarachnoid hemorrhage; SBP, systolic blood pressure; SD, standard deviation; TIA, transient ischemic attack; Tmax, the highest body temperature before MRI.

** *P* < .01.

### 3.2 Receiver operating characteristic curve analysis

**Figure 3:**
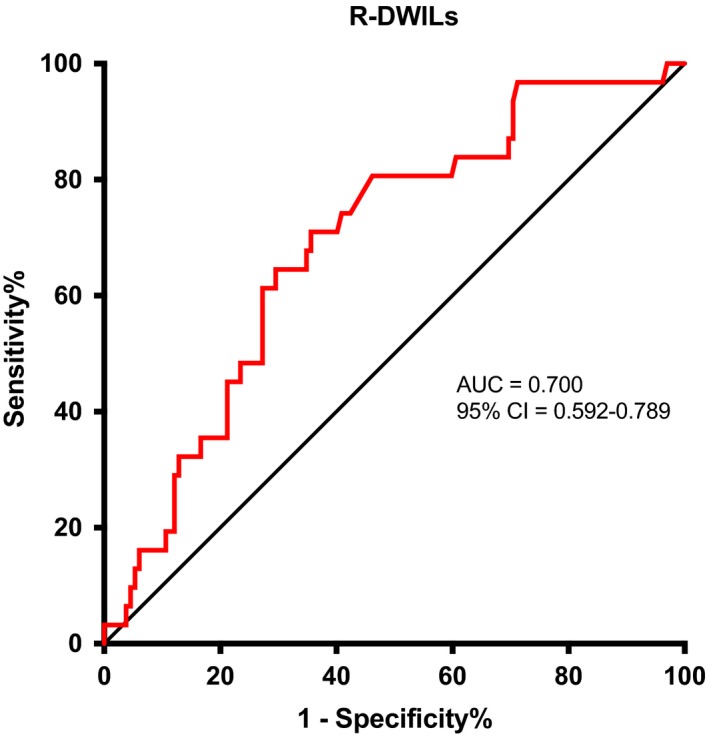
Receiver operating characteristic curves for R‐DWILs in patients with spontaneous ICH. Area under the curve was 0.700 (95% CI 0.592‐0.789; *P* = .001) for NLR. The Youden J index is the difference between the true‐positive rate and the false‐positive rate. Maximizing this index allows the identification of an optimal cut‐off point of 6.01 independently

The results and AUCs were shown in Figure 3. Based on the ROC curve, the best optimal cutoff value of NLR was projected to be 6.01 at 24‐48 hours after ICH onset, which yielded a sensitivity of 71% and a specificity of 64.4%, with the area under the curve at 0.700 (95% CI 0.592‐0.789; *P* = .001).

### 3.3 Frequency and other inflammatory markers with elevated NLR after ICH

In this cohort of patients with spontaneous ICH, we found that NLR was elevated in approximately 43.5% (71/163) of patients. In the univariate analysis, patients with elevated NLR had a median GCS of 13^10^, ^11^, ^12^, ^13^ and a median NIH stroke scale of 9^4^, ^5^, ^6^, ^7^, ^8^, ^9^, ^10^, ^11^, ^12^, ^13^ when compared with patients without elevated NLR (GCS: 15 [14‐15], *P* = .001; NIH stroke scale: 6 [3‐10], *P* = .005). In addition, those patients were prone to have a higher parenchymal hematoma volume and IVH extension. Of note, other inflammatory markers, such as body temperature (median, 38.2 vs 37.7, *P* < .001), ESR (median, 14 vs 12, *P* = .013), hs‐CRP (median, 11.9 vs 4.9, *P* < .001), and GLU (median, 6.6 vs 5.4, *P* < .001), were calculated significantly higher in patients with elevated NLR (Table 2).

**Table 2:** Comparison of inflammatory markers of patients by the cutoff value of NLR at 24‐48 h after ICH

	NLR ≤ 6.01 (n = 92)	NLR > 6.01(n = 71)	*P*‐value
Glasgow coma scale, median [IQR]	15 [14, 15]	13 [10, 13]	.001**
NIH stroke scale, median [IQR]	6 [3, 10]	9 [4, 13]	.005**
Hematoma volume, cm^3,^ median [IQR]	10.1 [5.0, 18.3]	15.1 [6.7, 27.8]	.014*
Tmax before MRI, °C, median [IQR]	37.7 [37.4, 38.0]	38.2 [37.8, 38.5]	<.001***
ESR, mm/h, median [IQR]	12.0 [6.3, 17.8]	14.0 [10.0, 21.0]	.013*
hs‐CRP, mg/L, median [IQR]	4.9 [3, 7.7.0]	11.9 [5.5, 45.1]	<.001***
GLU, mmol/L, median [IQR]	5.4 [5.0, 6.4]	6.6 [5.8, 7.2]	<.001***
Fibrinogen, g/L, median [IQR]	3.3 [2.8, 3.9]	3.5 [3.0, 4.3]	.070
IVH (n %)	21 (22.8)	36 (50.7)	<.001***

Abbreviations: ESR, erythrocyte sedimentation rate; GLU, fasting blood glucose; NLR, neutrophil‐to‐lymphocyte ratio; hs‐CRP, high sensitive c‐reactive protein; IVH, intraventricular hemorrhage; R‐DWILs, remote diffusion‐weighted imaging lesions; Tmax, the highest body temperature before MRI.

* *P* < .05.

** *P* < .01.

*** *P* < .001.

### 3.4 Association between elevated NLR and R‐DWILs

Table 3 summarized the results of the multivariate binary logistic regression of the R‐DWILs. With adjustment for age, sex, atrial fibrillation, previous ischemic stroke/TIA, SBP on admission, hematoma volume, and IVH, elevated NLR indicated a 3.17‐fold increased risk (OR = 3.170, 95% CI 1.306‐7.697, *P* = .011) of R‐DWILs. We furtherly explored the definite relationship with binary logistic regression analysis between leukocytes and R‐DWILs; however, the leukocytes obtained at 24‐48 hours after ICH onset were not independently associated with R‐DWILs.

**Table 3:** Multivariable analysis for association factors of R‐DWILs in patients with spontaneous ICH

	R‐DWILs	
	OR (95% CI)	*P*‐value
Model 1		
Age	1.035 (1.002‐1.070)	.039
NLR > 6.01	3.655 (1.534‐8.709)	.003
Model 2		
NLR > 6.01	3.170 (1.306‐7.697)	.011
IVH	2.944 (1.256‐6.900)	.013

Note

Model 1: bivariate logistic regression analysis with adjustment for age and sex; Model 2: bivariate logistic regression analysis with adjustment for age, sex, atrial fibrillation, previous ischemic stroke/TIA, SBP on admission, hematoma volume, IVH, and NLR.

## 4 DISCUSSION

In this study, we evaluated the circulating NLR in a cohort of 163 patients with spontaneous ICH who presented to our department of neurology. Here, we showed that there was an increased NLR level in patients with R‐DWILs at 24‐48 hours after ICH onset. The best discriminating value of NLR for R‐DWILs was 6.01, which was associated with a 3.17‐fold risk for R‐DWILs.

Since the time interval was critical for the occurrence of ischemic lesions associated with ICH,^11^ we had the strength that all patients underwent MRI imaging at 5‐8 days after ictus. R‐DWILs were detected among 19% of patients with spontaneous ICH in our cohort. Our results were corroborating with the prevalence reported in previous studies, ranging from 11.1%‐41%.^6^, ^7^, ^8^, ^9^ Here, we provide further evidence that R‐DWILs occur frequently, which therefore require our further attentions. Past studies linked R‐DWILs to aggressive early blood pressure lowering,^8^, ^9^ remote extension of hematoma,^20^ or the progression on cerebral microangiopathy.^7^, ^11^, ^21^, ^22^ However, the underlying mechanisms of R‐DWILs remained uncertain.

The inflammatory reactions following acute hemorrhagic stroke occur rapidly and typify a highly intricate interaction between the resident cells in the brain and those in the peripheral immune systems.^23^, ^24^ Previous experimental and clinical evidence supported that neuroinflammation played an essential role in the secondary brain injury after ICH and may lead to deleterious outcome.^25^ Circulating leukocytes were elevated immediately after ictus, which reflects the inflammation activation at some extend. Therefore, several studies implied that leukocytes could be involved in the pathogenesis of R‐DWILs after ICH. In a post hoc analysis of ERICH study,^8^ Kidwell, et al found that patients who developed ischemic lesions showed a higher circulating leukocyte counts; and after controlling for confounding factors, there was a trend for an association between them (OR = 1.050; 95% CI 0.993‐1.111; *P* = .084). Whereas in another prospective study enrolled 97 patients with acute hypertensive ICH, the circulating leukocytes on admission did not differ between patients with or without ischemic lesions.^11^ This inconsistency between the findings from these studies and those in our patients might be explained by the lack of strict exclusion criteria to control confounding factors, like infections. The relatively small sample size in these studies may be another reason for concealing the association between ischemic lesions and circulating leukocytes. Moreover, since they analyzed leukocytes as the target entity, the changes within different subtypes of leukocyte were neglected. Thus in the present study, we rigorously evaluated the NLR levels in the patients with ICH and excluded the infections and other comorbidities, which might lead to bias.

Peripheral sterile inflammatory response has been described in the context of stroke‐induced stress reaction, which usually is accompanied by neutrophils demarginated, activation,^26^, ^27^, ^28^ and lymphocytes apoptosis.^29^, ^30^, ^31^ NLR integrates both innate immune response (represented by neutrophils) and adaptive immune response (represented by lymphocytes), and furthermore, it also reflects the shift between them. Therefore, the NLR is superior to the merely sum of leukocytes or neutrophils or lymphocytes in estimating individual inflammatory status. In patients with spontaneous ICH, elevated NLR has been closely related to the risk of mortality and 90‐day morbidity,^16^, ^17^ and it also associated with stroke severity and peri‐hematoma edema growth.^15^, ^32^ The recent remarkable observations indicated that NLR was an independent predictor for delayed cerebral ischemia in patients with a SAH,^33^ making it a prime candidate for assessing the inflammatory aspects of ICH.

Here, we found that NLR was independently associated with the occurrence of R‐DWILs. The roles of neutrophils in promoting blood coagulation and thrombosis may enhance the occurrence of ischemic lesions. Neutrophils originated from the blood circulating could be found within the brain as early as 4 hours in mice ICH models.^34^ And the results from the literature suggest that neutrophils could externalize decondensed nucleosomes and granule proteins to form neutrophil extracelluar traps (NETs) in some cases.^35^ In this context, with the pre‐existing cerebral microangiopathy in ICH, elevated NLR was prone to have NET increasing and thus led to microvascular thrombosis. Another potential mechanism could be the NET‐induced activation of both the extrinsic and contact pathway of blood coagulation.^35^, ^36^ In our cohort, the higher NLR group showed a trend for higher fibrinogen accumulation in peripheral. A recent study found that patients with elevated neutrophil counts had a lower risk of hematoma expansion following acute ICH,^37^ may also be explained by the increased coagulation by neutrophils. These findings suggested that higher NLR indicated a tendency of coagulation or forming of microembolism. This might explain the relationship between high NLR and R‐DWILs.

Besides elevated NLR, the presence of IVH was the other correlated factor of R‐DWILs in our cohort. Analyzing the CSF in patients with IVH showed an early and modest elevation in leukocytes.^38^ IVH induced the blood‐brain barrier disruption, the more rigorous immune alterations in CSF, and the extension of hemoglobin degradation product might contribute to the presence of the higher R‐DWILs and NLR. Still, the mechanisms underlie in R‐DWILs in IVH requires further study.

Some limitations of this study should be noted. First, the generalizability of our conclusions is limited by our single‐center retrospective study design. Second, the low in‐hospital mortality in relation to our ward‐based approach and a low proportion of patients with ICH enrollment due to the strict inclusion and exclusion criteria might add selection bias into the analysis. Third, the clinically unstable cases cannot tolerate an MRI examinations are excluded, and therefore, there is likely to introduce bias toward patients with less severe ICH as well. Forth, we excluded patients with active infections prior to stroke or with early infection signs on admission. Inevitably, we cannot rule out those with only subclinical symptoms. Moreover, we had no detailed microbiological and viral data confirming potential infections occurring before. Fifth, there were no serial MRI scan data within our cohort, which makes it possible that R‐DWILs presented before or concurrently with ICH onset and had reversed by the time of undergoing MRI; the possibility also existed that patients without R‐DWILs in our study might present later. Finally, we neither confirmed these remote hyperintense lesions on DWI were truly cellular infarction nor investigated prothrombotic mediators, such as NETs, indeed elevated in those patients with R‐DWILs. These will be the key points in our following work.

In conclusion, the current study showed that the elevated NLR at 24‐48 hours was associated with the presence of R‐DWILs. Our findings highlighted the role of neutrophils as indicator of thromboinflammory reaction in ICH. From the practical point of view, patients with a high NLR at 24‐48 hours should be intensely noted for the increased risk of R‐DWILs. This may assist patient care and may be useful in selecting patients for DWI scans and thus improving clinical outcome.

## CONFLICT OF INTEREST

The authors declare no disclosures or conflicts of interest.
